# Bioactive Peptides in Dairy Milk: Highlighting the Role of Melatonin

**DOI:** 10.3390/biom14080934

**Published:** 2024-08-01

**Authors:** Melania Andrani, Eleonora Dall’Olio, Fabio De Rensis, Padet Tummaruk, Roberta Saleri

**Affiliations:** 1 Department of Veterinary Science, University of Parma, Via del Taglio 10, 43126 Parma, Italy; eleonora.dallolio@unipr.it (E.D.); fabio.derensis@unipr.it (F.D.R.); roberta.saleri@unipr.it (R.S.); 2 Centre of Excellence in Swine Reproduction, Department of Obstetrics, Gynecology and Reproduction, Faculty of Veterinary Science, Chulalongkorn University, Bangkok 10330, Thailand; padet.t@chula.ac.th

**Keywords:** melatonin, dairy cow, milk, milking time

## Abstract

Melatonin, an endogenous indolamine derived from tryptophan, is primarily synthesized by the pineal gland in mammals and regulated by a complex neural system. Its release follows a circadian rhythm, which is crucial for regulating physiological processes in response to light–dark cycles in both humans and animals. In this review, we report that the presence of this hormone in bovine milk, with significant differences in concentration between daytime and nighttime milking, has increased interest in milk as a natural source of bioactive molecules. Melatonin lowers cortisol levels at night, reduces body temperature and blood pressure, coinciding with decreased alertness and performance, acts as an antioxidant and anti-inflammatory agent, modulates the immune system, offers neuroprotective benefits, and supports gastrointestinal health by scavenging free radicals and reducing oxidative stress in dairy cows. Many factors influence the release of melatonin, such as the intensity of artificial lighting during nighttime milking, the frequency of milkings, milk yield, and genetic differences between animals. Nocturnal milking under low-intensity light boosts melatonin, potentially reducing oxidative damage and mastitis risk. Additionally, ultra-high temperature (UHT) treatment does not significantly affect the melatonin content in milk. However, further research on its stability during milk processing and storage is crucial for ensuring product efficacy. In some countries, nighttime milk with naturally elevated melatonin content is already commercialized as a natural aid for sleep. Thus, naturally melatonin-rich milk may be a promising alternative to synthetic supplements for promoting better sleep and overall well-being.

## 1. Biological Characteristics

**Figure 1:**
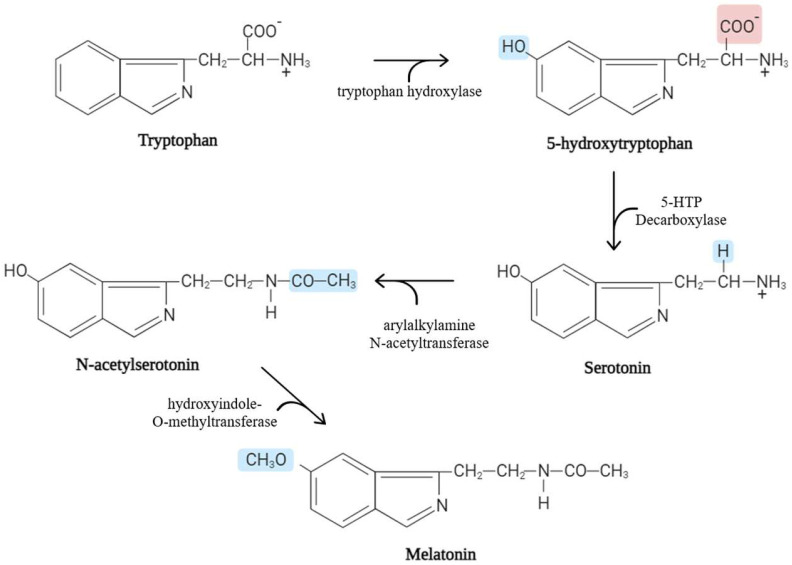
Biosynthetic melatonin pathway from tryptophan.

Melatonin (C_13_H_16_N_2_O_2_), an indolamine derived from tryptophan and synthesized by the pineal gland in mammals [1], shares its origin with serotonin, with a molecular weight of 232.2 Dalton. Melatonin is an ancient molecule with high conservation across various species. It is an amphiphilic molecule, featuring hydrophilic and lipophilic components, which facilitate its direct passage into cells. Tryptophan is first converted to 5-hydroxytryptophan by tryptophan hydroxylase (TPH), which is further transformed into serotonin. Subsequently, serotonin undergoes acetylation by arylalkylamine N-acetyltransferase (AANAT), forming N-acetylserotonin (NAS). The last step involves the conversion of NAS to melatonin, facilitated by acetylserotonin O-methyltransferase (ASMT), also known as hydroxyindole-O-methyltransferase (HIOMT) [2] (Figure 1).

The AANAT enzyme limits the production of melatonin as its activity is minimal during the day, allowing, on the one hand, the accumulation of serotonin in pinealocytes and, on the other hand, a minimal conversion to melatonin. The activity of the enzyme increases with the onset of darkness leading to melatonin synthesis [3].

A multisynaptic pathway connects the central nervous system to the epiphysis, with sympathetic nerve fibers releasing norepinephrine (NE) exclusively at night. NE, the main neurotransmitter in regulating AANAT, triggers melatonin synthesis in mammals [4,5]. Activation of β1-adrenergic receptors increases cyclic AMP, essential for AANAT stimulation. Additionally, the activation of α1-adrenergic receptors increases intracellular calcium concentration ([Ca^2+^]), whose role in melatonin regulation varies across species [6,7].

### Regulation of Melatonin Release

Melatonin release begins within six months of birth in humans, maintaining a circadian rhythm until middle age. However, in both humans and animals, aging leads to reduced nighttime melatonin levels due to factors like decreased gland innervation, adrenergic receptor decline, and gland calcification, as observed in studies by Tan et al. [8] and Peruri et al. [9].

In mammals, unlike typical endocrine glands, the pineal gland does not accumulate melatonin [10]. Melatonin, released exclusively at night into the bloodstream, synchronizes physiological processes and impacts the sleep–wake cycle (“circadian rhythms”), highlighting its pivotal role in maintaining overall biological balance and rhythm [11].

The pineal melatonin binds to albumin and is promptly released into the bloodstream and the cerebrospinal fluid (CSF), thereby permeating various regions of the CNS and all peripheral organs [12]. In these target locations, diverse effects are elicited through distinct mechanisms of action. The half-life of melatonin in the bloodstream is approximately 30 min in cows [13], as it undergoes conversion to 6-hydroxymelatonin by cytochrome P450 isoforms, monooxygenases (mainly CYP1A2), and subsequent conjugation to 6-sulfatoxymelatonin in the liver [14] and kidneys, facilitating urinary excretion [15]. There are two mechanisms by which melatonin is eliminated: hydroxylation and oxidation [11].

The suprachiasmatic nucleus (SCN) acts as the central pacemaker, receiving light cues via the retina-hypothalamic pathway. In response, the SCN modulates hormone secretion from the pituitary gland and melatonin release from the pineal gland. This intricate regulation, described by Hastings et al. [16] and von Gall et al. [5], underscores the pivotal role of the circadian timing system in coordinating physiological processes, crucial for maintaining the body’s internal clock and synchronizing with external environmental changes.

**Figure 2:**
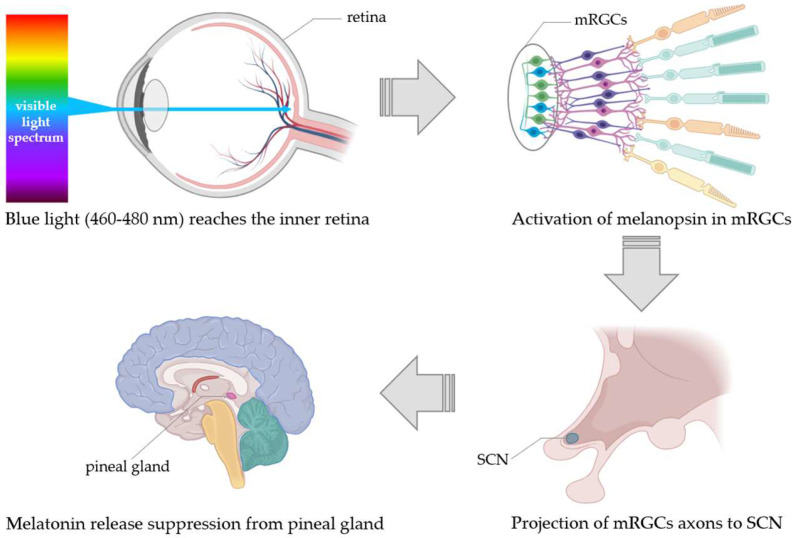
Representation of the suppression of melatonin release after light activation of melanopsin contained in mRGCs. mRGCs = melanopsin retinal ganglion cells; SCN = suprachiasmatic nucleus.

A small group of specialized retinal cells, comprising about 1–2% of total retinal neurons, are crucial for detecting and transducing blue light wavelengths (460–480 nm) that inhibit melatonin synthesis [17,18,19]. These melanopsin retinal ganglion cells (mRGCs) contain the pigment melanopsin, and their axons project directly to the suprachiasmatic nucleus (SCN) [20]. mRGCs are vital not only for melatonin regulation but also for non-image-forming functions such as sleep regulation and circadian photoentrainment (Figure 2) [21]. While rods and cones can partially compensate, the absence of mRGCs leads to a total loss of these light-dependent responses. Melanopsin mediates blue light signal transduction, and its activation suppresses sleep induction, while in melanopsin knock-out mice, sleep induction improves [22]. Upon light activation, melanopsin triggers a G-protein cascade, leading to membrane depolarization [23]. Melanopsin function decreases before melatonin onset, reaching its lowest after melatonin begins, independently of external circadian signals [24]. Daytime blue light exposure is crucial for activating melanopsin, modulating circadian-related genes, and suppressing melatonin to maintain alertness and cognitive performance [25]. Conversely, blue light exposure before bedtime can disrupt sleep quality and circadian rhythms [26].

## 2. Roles and Mechanisms of Action

The main role of melatonin is the regulation of the sleep–wake cycle [11]. It is responsible for suppressing the wake-promoting signal of the circadian clock in humans and other diurnal species at the SCN, which promotes sleep. Moreover, the default mode network regions in the brain are affected by melatonin to promote fatigue and sleep-like changes [27].

Melatonin regulates hormone secretion, particularly inhibiting hypothalamic corticotropin-releasing hormone (CRH), which lowers adrenocorticotropic hormone (ACTH) and cortisol levels at night [28]. Increased melatonin at night also reduces body temperature and blood pressure, coinciding with decreased alertness and performance; these effects are mediated through the hypothalamus [29]. Additionally, melatonin acts as an antioxidant and anti-inflammatory agent, modulates the immune system, offers neuroprotective benefits, and supports gastrointestinal health by scavenging free radicals and reducing oxidative stress [30,31,32,33]. As a lipophilic molecule, melatonin can cross the blood-brain barrier, performing actions both with and without receptor involvement. Receptor-independent actions include directly scavenging radicals or acting on nearby cells, adding complexity to their physiological roles [34]. Its receptor-mediated actions involve interactions with various intracellular targets, such as transporters, ion-binding proteins, enzymes, cytoskeletal components, and mitochondria [35]. Conversely, receptor-independent actions involve donating electrons to free radicals, which detoxifies them, stimulates antioxidative enzymes, inhibits prooxidative enzymes, promotes glutathione synthesis, and reduces free radical generation by improving mitochondrial function. These dual mechanisms highlight melatonin’s multifaceted role in maintaining physiological balance and protecting cellular health [36].

### 2.1. Receptor-Mediated Action

Melatonin receptors are present in various tissues, such as the retina, brain, kidneys, gastrointestinal tract, skin, immune, endocrine, reproductive, and cardiovascular systems. There are two intramembrane (MT1 and MT2), a cytosolic (MT3), and a nuclear (ROR/RZR) receptors [37].

The MT1 (350 amino acids) and MT2 (362 amino acids) are G-protein-coupled receptors (GPCR), characterized by 4-intracellular and 4-extracellular domains and 7-transmembrane-helices, that exert distinct roles [35].

Melatonin exhibits a greater affinity for MT1, which is intricately linked to circadian rhythms and sleep regulation, compared to MT2. MT1 receptors are expressed in various tissues, including the cardiovascular system, skin, pancreas, liver, spleen, adrenal cortex, ovary, placenta, and breast. MT1 mRNA expression displayed an evident circadian pattern, reaching its peak during the subjective night phase. Notably, the activation of MT1 receptors is essential in facilitating melatonin sleep-promoting effects [38]. MT1 also becomes of interest from the perspective of potential treatments for sleep disorders since it also mediates the melatonin function in the phase shifting of circadian rhythms [39].

MT2 receptors are present in the brain, adipose tissue, blood vessels, mammary glands, and gastrointestinal tract [40]. Melatonin binds to MT1 and MT2 receptors, inhibiting the AC/cAMP/PKA/CREB pathway, activating calcium signalling, and regulating hormone synthesis. It activates the Rafs-MEK1/2-ERK1/2 pathway for cell proliferation control and ERK-MAPK/JNK for oxidative stress management. Melatonin also triggers the PI3K/Akt pathway for cardio protection, inhibiting tumour cell proliferation via mTOR and apoptosis modulation, while inhibiting the GC/cGMP/PKG pathway [41].

Melatonin also binds with lower affinity to MT3, which acts as a quinone reductase 2 (QR2) and can neutralize free radicals. MT3 binding is temperature dependent; when the temperature reaches 37 °C, neither melatonin nor the so-called specific MT3 receptor agonist, MCA-NAT, binds to the MT3 site. By contrast, temperature does not influence melatonin binding to the MT1 and MT2 melatoninergic receptors [42].

The ROR (retinoic acid receptor-related orphan receptors) family, akin to melatonin diverse functions, influences numerous physiological processes. Studies reveal reciprocal interactions between RORs and melatonin, with melatonin notably enhancing RORα-mediated transcriptional activity. This melatonin-RORα axis impacts immunity, reproduction, cardiovascular function, oxidative stress, circadian rhythms, development, and oncogenesis [37,43].

### 2.2. Non-Receptor-Mediated Actions

Melatonin exerts various non-receptor-mediated actions, such as antioxidant properties, involving scavenging free radicals and reducing oxidative stress in cells and tissues. Through its antioxidant effects, melatonin can help protect against cellular damage and aging processes caused by reactive oxygen and nitrogen species [37].

Additionally, melatonin modulates the immune system by stimulating immune cells and regulating cytokine production, which enhances immune function and helps mitigate excessive immune responses seen in autoimmune diseases [44].

Melatonin inhibits the production of pro-inflammatory molecules and signaling pathways involved in the inflammatory response. By dampening inflammation, melatonin may alleviate symptoms associated with various inflammatory conditions, including autoimmune diseases and neurodegenerative disorders. In the neuroprotection area, melatonin plays a crucial role by protecting neurons against damage and cell death. Its neuroprotective effects stem from its antioxidant and anti-inflammatory actions, as well as its ability to modulate neurotransmitter systems. These properties make melatonin a promising candidate for the prevention and treatment of neurodegenerative diseases [45].

Moreover, melatonin contributes to gastrointestinal health by maintaining mucosal integrity, regulating motility and secretion, modulating gut inflammation, supporting digestive function, and managing gastrointestinal disorders [46].

In reproductive physiology, melatonin regulates gonadal activity, synchronizes reproductive processes with circadian rhythms, and influences reproductive hormones, gamete production, and fertility [15].

In summary, after reporting the most important metabolic and functional characteristics of melatonin, this study aims to focus on and highlight the relationship between melatonin content in dairy cow milk and the natural or artificial photoperiod, with a mention of the effect of milking frequency on melatonin production.

## 3. Melatonin in Bovine Milk

In dairy cows, similarly to humans and goats, melatonin is released into milk [47,48] and its concentrations reflect the circadian rhythm of production. Therefore, the highest concentration of melatonin in milk is produced during the night and milked in the early hours of the morning [49,50,51,52,53]. Table 1 summarizes the available data regarding melatonin content in nighttime and daytime in dairy milk. In addition, it should be emphasized that the studies on melatonin content in bovine milk currently available are few, and the differences in the absolute values of concentration of melatonin observed between studies are influenced by several factors, such as seasonality, the latitude, the genetics of animals, the milk yield, and the number of milkings.

The composition of milk (even melatonin quantity) varies due to several factors, mainly nutritional and physiological. Available studies report that melatonin concentrations in milk exhibit significant diurnal variations, being higher during the night (Table 1). Sahin et al. [54] in Turkey found melatonin levels of 163.13 ± 8.96 pg/mL at night and 103.70 ± 6.61 pg/mL during the day. Teng et al. [53] in China reported that melatonin levels in milk were higher at night (120.07 pg/mL) compared to daytime milk (90.21 pg/mL) during the warm season.

Environmental, photoperiod, climate changes, and temperature play a role in the seasonal changes in melatonin production. Several studies confirm that the release of melatonin is greater in night milking; it is also observed that the concentration is greater during the winter seasons compared to the summer ones.

In fact, in summertime (August), Kollmann et al. [50] reported that in Germany, melatonin levels in cow milk ranged from 1.8 to 4.4 pg/mL during the evening milking and between 3.9 and 6.7 pg/mL during the morning milking. Similarly, Castro et al. [55] found that in June (in Spain), the trend of melatonin concentration in milk paralleled that in blood, with lower levels in milk (at 01:00 AM: blood: 25.4 ± 5.6 pg/mL; milk: 2.9 ± 0.6 pg/mL). On the contrary, Asher et al. [56], in wintertime (November) in Israel, showed that the presence of greater hours of darkness leads to higher melatonin concentrations than in summer (“Night-Milk” was 30.70 ± 1.79 pg/mL compared to “Daily-Milk” at 17.81 ± 0.33 pg/mL).

Even the genetic difference between breeds or species can influence milk concentration of melatonin. Boztepe et al. [57] highlights a significant difference between the levels of melatonin in the daytime and nighttime milk of Holstein and Jersey cows; in Holsteins, the melatonin was 2.912 pg/mL and 11.314 pg/mL, compared to Jersey cows at 2.924 pg/mL and 6.954 pg/mL (day and night respectively). Erikson et al. [47] on the other hand, studied that the distinction between cows and goats was in the volume of distribution and the steady-state distribution volume, both of which were greater in cows. In addition, the concentration differences could be due to genetic differences, as has been reported in ewes, where seasonal melatonin concentrations (June and December) were shown to have a heritability of h2 = 0.45 [58].

The administration of naturally melatonin-rich cow milk (night milking) in Wistar rats can increase plasma melatonin levels by 26.5%, and if tryptophan is added in this milk, the increase in blood melatonin rises to 35.5% [52]. Similarly, in humans, positive effects of consuming milk naturally rich in melatonin have been demonstrated, both on the improvement of sleep and anxiety states [59,60,61]. Valtonen et al. [59] found that consuming 0.6 L of melatonin-rich milk daily improved sleep and daily activity.

Among the parameters to consider that are related to the production of each cow, in high-production animals, the melatonin content is diluted in a greater volume of milk. As reported by Romanini et al. [62], the higher milk melatonin levels in low-producing animals compared to high-producing ones came from milk collected during winter, while the lowest concentration was found in the daytime milk in the high-production group. This study also highlighted that the concentration of melatonin in milk can be significantly influenced by luminance variables, seasonality, and the time of milking.

Finally, the milking frequency modifies the yield and composition of milk [63,64] and increasing the frequency of milking from twice to four times daily boosts overall milk production by altering mammary gland gene expression [65]. In a recent study, it has been observed that increasing milking frequency at night raises milk melatonin levels [66]. However, Helmreich et al. [67] conducted a study on 125 cows from eight different automated milking system farms in Switzerland and revealed a correlation between increased nocturnal milkings and decreased salivary melatonin levels, suggesting a potential disruption in the cow’s circadian rhythm. In this study, as the number of milkings increased and the cow’s exposure to light enhanced, there was a decrease in melatonin synthesis. Moreover, the study hinted at a relationship between milking frequency, exposure to artificial light, and cortisol production.

To better understand the mechanisms of melatonin release between night and day, studies were carried out on the effect of artificial light in stables. Artificial light at different intensities can affect the production of melatonin in dairy cow [68,69,70] and it has also been found that the increase in duration of the dark phase and the use of low-intensity lights instead of high-intensity lights at night determines an increase in the melatonin content in nighttime milk in humans [71] and dairy cows [56]. Furthermore, Asher et al. [72] explored the impact of lighting conditions on milk composition in cows under natural light cycles during the warm season (3.7 months, >28 °C) and cold season (3.3 months, <20 °C), and the results showed no significant differences in milk yield or composition (fat, protein, and lactose) between the groups. However, milk from the Night-Dark group exhibited notably higher melatonin content compared to the Night-Illuminated group, suggesting the concept of “Chrono-functional milk”. Additionally, the Night-Dark group’s milk showed significantly lower somatic cell counts (SCC), indicating potential benefits for milk quality and cow health by reducing mastitis risk. Melatonin presence was negatively correlated with SCC, highlighting melatonin’s role in enhancing neutrophil protection against oxidative damage, thus underscoring melatonin’s potential in improving milk quality and cow welfare. Therefore, it could be of great interest to dairy farms to apply changes in lighting, with a simple switch from high- to low-intensity lights, to improve mastitis resistance and to obtain a milk that is naturally rich in melatonin.

Considering the effect of blue visible light on melanin activity in human milk, a lower concentration of circulating melatonin has been reported following exposure to monochromatic blue LED light [73,74]. In a following study, Elsabagh et al. [75] compared the impact of LED monochromatic blue light versus yellow light on melatonin production in female dairy calves and observed a partial suppression of melatonin synthesis during the hours of treatment. Furthermore, it emerged that a greater blood concentration of nocturnal melatonin was observed in female calves subjected to yellow light (23.7 ng/mL) compared to female calves exposed to blue light (18.3 ng/mL). However, the greater sensitivity to lower intensity blue LED light found in this study could be due to the younger age of the experimental animals. The concentration of melatonin can be increased by different approaches, for example, by increasing the period in which the animals remain in the dark, by exposing the animals to light at different wavelengths during the day and night [76], or by setting the maximum illumination to no more than 50 lux (up to 35 pg/mL) [77].

Once it has been clarified what influences the release of melatonin, interest will be dictated by the fact that the effects of its supplementation on dairy cow, by subcutaneous implants or by rumen bypass melatonin feeding, induces a decrease in SCC in the milk of cows with both clinical and subclinical mastitis [78,79]. Melatonin can have a potential protective role against thermal stress. Teng et al. [53] investigated the levels of HSP70 and HSP90 (heat-shock protein) in night milk and found that it was reduced. This effect might be related to the higher concentration of melatonin in nighttime milk. Because heat stress is a predominant problem for dairy farms in the warm season of the year, the author suggested that melatonin can provide valuable insight into the physiological responses of dairy cows under seasonal heat stress.

Supplementation of melatonin also seems to have a positive effect on fertility because during the warm season, in cows under heat-stress conditions, melatonin administration induces a reduction in embryonic losses [80] and an increase in pregnancy rates [81] in dairy cows’ undergoing timed artificial insemination (TAI protocol).

In small ruminants, such as sheep and goats, melatonin is used to enhance their reproductive capacity. Studies show that out-of-season melatonin application positively impacts reproductive performance in sheep and goats by increasing progesterone synthesis, enhancing implantation and embryo survival, and increasing the stimulated follicle number and the occurrence of multiple offspring [82,83]. Melatonin implants can boost libido in males and induce estrus in females during or outside the breeding season [83,84,85]. In addition, the exogenous melatonin can lead to a reduction in milk production [86]; nevertheless, it has a positive effect on milk quality and animal health by reducing the SCC [48,87].

However, when melatonin is supplemented in cows, the fact that melatonin supplementation reduces prolactin levels and therefore milk yields in grazing cattle must be considered [88]. Furthermore, the release is influenced by different parameters, but it is interesting to note that there are no significant differences in melatonin content between bulk tank milk, UHT milk, and day milk collected in summer [62]. This suggests that the UHT process does not significantly alter the melatonin content. This finding is significant because it implies that supply chain treatments, particularly the UHT process, may not affect the concentration of melatonin in milk.

Therefore, melatonin levels in milk were influenced by weather, season, milk production, and milking time. Night milk had higher melatonin (average 14.87 pg/mL) than day milk (average 6.98 pg/mL). Winter milk had more melatonin than summer milk, in which low-production winter night milk had the highest concentration (41.94 pg/mL) [62].

These findings suggest benefits for marketing melatonin-rich milk. Several countries, such as Ireland, New Zealand, Finland, and the United States, were considering marketing it. This could have important implications for the dairy industry, as it opens new opportunities for product differentiation and marketing. However, it is crucial to conduct further studies to confirm that all supply chain treatments do not influence melatonin levels. Comprehensive research in this area would ensure that consumers receive consistent product quality, and it would help establish standardized processing methods that preserve melatonin content in milk.

**Table 1:** Summary of the average data reported in the cited studies referring to “night milk” and “day milk”. ND = Night-Dark group; NI = Night-Illuminated group.

Breed	Lactation Stage	Milk	Technique for Melatonin Detection	Melatonin (pg/mL)		Lighting (h)		Place	References
Animals (n°)	Days after Calving	kg/day		Night Milk	Day Milk	Light	Dark	Maps Coordinate	
Holstein (30)	/	23	ELISA KIT RE54041 (IBL International, Hamburg, Germany)	14.9 ± 7.7	6.9 ± 3.1	10.30 winter 13.30 summer	Cycle of 2 h light on, 5 h light off, 5 h light on	Castro, Brazil (24°49′06.7″ S 50°00′54.1″ W)	[62]
Holstein (10)	100–150	25	ELISA KIT RE54041 (IBL International, Hamburg, Germany)	39.4	4.0	/	/	Viçosa, Brazil (37°52′21.0″ N 32°28′28.1″ E)	[52]
Holstein (40)	/	/	ELISA KIT MBS743340 (MyBiosource, California, USA)	163.1 ± 8.9	103.7 ± 6.6	/	1-week total darkness	Konya, Turkey (37°52′18.5″ N 32°29′32.5″ E)	[54]
Holstein (10)	150 ± 20	25 ± 5	UHPLC	120.1	90.2	13 (August)	11 (August)	China (34°48′08.5″ N 113°41′13.1″ E)	[53]
Holstein (28)	135	34.5	ELISA KIT RE54041 (IBL International, Hamburg, Germany)	30.7 ± 1.8 (ND) 17.8 ± 0.3 (NI)	5.4 ± 0.3 (ND) 3.3 ± 0.2 (NI)	10.40 (November)	13.60 (November)	Israel (32°42′24.0”N 35°10′46.6″ E)	[56]

## 4. Conclusions

The melatonin circadian rhythm is maintained in dairy cows, with significant differences in melatonin content between daytime and nighttime milk. Research indicates several potential benefits of melatonin for milk quality and cow health. High melatonin concentrations in milk are associated with protective effects against thermal stress, as evidenced by reduced heat-shock protein levels in nighttime milk.

As described in the review, variations in melatonin levels are influenced by geographical latitude, environmental conditions, milking frequency, and exposure to artificial light [50,53,55,57,75]. Studies confirm that nocturnal milking under low-intensity lighting increases melatonin content, potentially enhancing milk quality and cow health due to melatonin’s role in reducing oxidative damage and lowering the risk of mastitis, as indicated by its negative correlation with somatic cell count [72].

Therefore, adjusting lighting from high to low intensity or increasing nighttime milking could benefit cow welfare by producing milk naturally rich in melatonin. This milk could improve the quality of human sleep when consumed. After all, our mothers have always known this. How many times have we heard this phrase? “And after dinner, a glass of steaming milk and then straight to bed”.

In some countries, such as Lullaby Milk in Ireland, Ingman Dairy’s Night Time Milk in Finland, Dreamerz in the United States, and iNdream3 in New Zealand, naturally melatonin-rich milk from nighttime milking is already marketed as a sleep aid [89].

However, there are no studies providing evidence of higher melatonin content in nighttime milk from high-production farms in temperate areas. Interestingly, the stability of melatonin through high-temperature processes like UHT treatment suggests its potential for enhancing the nutritional profile of commercial milk products [62].

In conclusion, a high melatonin level can improve cow health by reducing the risk of inflammation, such as mastitis, and increasing overall welfare. Furthermore, melatonin in dairy milk for human consumption can be beneficial as a natural sleep aid.
